# Racial disparities in patients diagnosed with light chain (AL) amyloidosis

**DOI:** 10.1038/s41408-021-00466-8

**Published:** 2021-04-10

**Authors:** Anita D’Souza, Liliana Pezzin, Purushottam Laud, Ashima Singh

**Affiliations:** 1 Department of Medicine, Medical College of Wisconsin, Milwaukee, WI 53226 USA; 2 Institute for Health and Equity, Medical College of Wisconsin, Milwaukee, WI 53226 USA; 3 Department of Pediatrics, Medical College of Wisconsin, Milwaukee, WI 53226 USA

**Keywords:** Cancer epidemiology, Myeloma

Dear Editor,

Light chain (AL) amyloidosis arises from a precursor plasma cell neoplasm that produces clonal free light chains that form insoluble fibril deposits leading to organ dysfunction^1^. Because the disease is rare, heterogeneous, and multi-systemic, it can take several months to years for the symptoms to show in patients before a diagnosis is made^2^. Knowledge of the pre-existing clinical characteristics of patients eventually diagnosed with AL amyloidosis is critical as it may inform early diagnosis of the disease. Evidence suggests that early diagnosis of AL amyloidosis leads to improved outcomes, including superior survival, as the disease can be modulated with recently available therapies^3,4^. Among diagnosed amyloidosis patients, Black men and women have the highest mortality rate^5^. Although existing literature has documented a racial predisposition in plasma cell disorders, such as monoclonal gammopathy of undetermined significance (MGUS) and multiple myeloma^6,7^, racial differences in the incidence of the multiple other potential clinical precursor diagnoses associated with AL amyloidosis remain unknown. In this report, we use nationally representative electronic health records (EHR) data to document and contrast the clinical characteristics of patients diagnosed with AL amyloidosis by self-reported race. We also compare the clinical characteristics between patients with AL amyloidosis and matched individuals without amyloidosis by race. We hypothesized that differences in AL-associated, pre-existing diagnoses will be evident in patients prior to their diagnosis of AL amyloidosis and that these differences will vary by the patients’ race.

Data for this analysis were drawn from TriNetX, a health research network providing access to high-quality de-identified patient-level data from EHR from large healthcare organizations. These data, which are refreshed on a regular basis, are made available through a research network that provides a HIPAA compliant platform with a built-for-purpose user interface and analytics capabilities. No protected health information or personal data is made available to the users of the platform. For this analysis, the TriNetX platform with browser-based real-time analytical features was used.

*Patient population*: Patients were coded to have AL amyloidosis if they (i) had two or more occurrences of diagnosis codes ICD10: E85.81, E85.4, E85.89, or E85.9 between Jan 1, 2010 and Dec 31, 2019 and (ii) received specific treatment after their AL amyloidosis diagnosis. Specific treatment included the use of one or more of the following: bendamustine, bortezomib, carfilzomib, cyclophosphamide, daratumumab, dexamethasone, doxorubicin, elotuzumab, etoposide, interferon alpha-2a/2b, ixazomib, lenalidomide, melphalan, panobinostat, pomalidomide, prednisone, prednisolone, thalidomide, vincristine, or transfusion of autologous hematopoietic cells. The sample was further restricted to individuals aged 40 or older due to the low incidence of the disease in younger adults. The cohort of AL amyloidosis patients was then stratified by race as African American/Black and White. The two comparison groups of Black and White patients without amyloidosis comprised individuals who (i) did not have any ICD code for AL diagnoses and (ii) had at least two visits with the healthcare system in the time period of Jan 1, 2010–Dec 31, 2019. To ensure the comparability of the comparison groups, we further restricted the cohorts to those who had at least one visit in 2019.

Pre-existing diagnoses were defined using diagnostic codes of interest present in an AL amyloidosis patient prior to the diagnosis of AL amyloidosis. These were grouped within four major categories: cardiac, renal, gastrointestinal/hepatic, other. For example, the cardiac category included diagnostic codes for cardiomyopathy, heart failure, cardiac arrhythmias, etc. The specific codes considered are provided in the tables.

*Statistical analyses*: We used *z*-tests/*t*-tests to compare the clinical characteristics of Black and White AL amyloidosis patients after matching for age, sex, and presence of type 2 diabetes. We matched with diabetes as some AL-associated diagnoses, e.g. cardiomyopathy, heart failure, proteinuria, chronic kidney disease, and neuropathy, may overlap with diabetes and there is a known racial difference in the prevalence of diabetic complications. We performed 1:1 matching based on the greedy nearest-neighbor matching algorithm using caliper of 0.1 pooled standard deviations (SD). The same methodology was used to compare the pre-existing clinical characteristics of patients with and without a diagnosis of AL amyloidosis by race.

A total of 4028 patients were identified as having a diagnosis of AL amyloidosis during the study period. Among Black patients with AL amyloidosis (*N* = 695), the mean age was 65 (SD 11) years with 55% males. The geographic distribution included 16% Northeast, 23% Midwest, 56% South, and 5% West. Among 3333 identified White patients, the mean age was 67 years (SD 11) with 58% male. The geographic distribution included 17% Northeast, 24% Midwest, 41% South, 17% West, and 1% unknown. The age difference by race appeared smaller in AL compared to MGUS^8^. After 1:1 matching, 691 Black AL patients matched with 691 White AL patients.

Compared to White AL patients, Black patients were more likely to have pre-existing MGUS as well as multiple other cardiac, renal, gastrointestinal AL-associated diagnoses, and carpal tunnel syndrome preceding the formal diagnosis of AL amyloidosis (Table 1). The difference in pre-existing MGUS was different (21% Black vs 13% White, *p* 4.48E−05) but pre-existing multiple myeloma was not different between the two groups (32.4% Black vs 30.83% White, *p* 0.53). This may suggest that once multiple myeloma is diagnosed, patients are in the care of specialists who may be vigilant for identification of amyloidosis and there is no racial difference in this diagnosis. The magnitude of differences was particularly striking for the diagnoses of cardiomegaly (28.51% vs 20.55%, *p* 0.0006), cardiomyopathy (37.19% vs 22.58%, *p* 2.93E−09), heart failure (54.85% vs 33.29%, *p* 6.85E−16), and chronic kidney disease (43.56% vs 35.02%, *p* 0.001), for Black compared to White patients, respectively. These clinical conditions are known harbingers of worse outcomes in AL amyloidosis. These findings raise concerns that Black patients with AL amyloidosis may be diagnosed later in the disease process. These findings also suggest that it may take longer or require a greater constellation of AL-associated symptoms/diagnoses before AL amyloidosis to be diagnosed in Black patients relative to White individuals. It is well known that patients with AL see multiple physicians including subspecialists for various diagnoses, e.g., proteinuria, carpal tunnel syndrome, cardiomyopathy, related to the amyloidosis diagnosis^2,9^, and our results suggest that race can be associated with AL amyloidosis diagnosis.

**Table 1:** Comparison of pre-existing diagnoses in AL amyloidosis by race.

ICD code	Pre-existing diagnosis	Black, with AL *N* = 691	White, with AL *N* = 691	*p*-value	SD
D47.2	MGUS	21.13%	12.88%	4.48E−05	0.22
C90	Multiple myeloma	32.42%	30.83%	0.53	0.03
***Cardiac diagnoses***					
I51.7	Cardiomegaly	28.51%	20.55%	0.0006	0.19
I42	Cardiomyopathy	37.19%	22.58%	2.93E−09	0.32
I49	Other cardiac arrythmia	30.25%	19.68%	5.7E−06	0.25
R06.0	Dyspnea	49.78%	35.17%	3.88E−08	0.30
R55	Syncope and collapse	13.46%	9.70%	0.03	0.12
R42	Dizziness and giddiness	14.33%	14.62%	0.88	0.01
R60	Edema	33.58%	26.77%	0.006	0.15
I50	Heart failure	54.85%	33.29%	6.85E−16	0.45
J90	Pleural effusion	11.43%	11.72%	0.87	0.01
I48	Atrial fibrillation and flutter	21.85%	21.85%	1	0
I95	Hypotension	21.71%	18.67%	0.16	0.08
***Renal diagnoses***					
N04,	Nephrotic syndrome	8.39%	9.84%	0.35	0.05
R80	Proteinuria	23.16%	19.97%	0.15	0.08
N18	Chronic kidney disease	43.56%	35.02%	0.001	0.18
***Neurological diagnoses***					
N52.9	Male erectile dysfunction	7.67%	4.34%	0.009	0.14
M79.2	Neuralgia and neuritis	2.17%	2.61%	0.60	0.03
R20	Disturbances of skin sensations	13.89%	11.00%	0.10	0.09
G60	Hereditary and idiopathic neuropathy	12.59%	10.42%	0.21	0.07
G62	Other and unspecified polyneuropathies	11.72%	11.29%	0.80	0.01
G90	Disorders of autonomic nervous system	2.75%	3.47%	0.44	0.04
***GI/Hepatic diagnoses***					
R13.1	Dysphagia	12.88%	9.70%	0.06	0.10
R16	Hepatomegaly and splenomegaly	5.79%	5.79%	1	0
K76	Other diseases of liver	13.17%	11.00%	0.22	0.07
R11	Nausea and vomiting	19.83%	16.35%	0.09	0.09
K59.0	Constipation	22.00%	14.76%	0.0005	0.19
R19.7	Diarrhea	15.63%	17.95%	0.25	0.06
R10	Abdominal pain	27.50%	19.25%	0.0003	0.20
***Other diagnoses***					
R53	Malaise and fatigue	30.83%	30.39%	0.86	0.01
G56.0	Carpal Tunnel Syndrome	11.14%	7.24%	0.01	0.14
D69	Purpura	15.20%	14.47%	0.71	0.02
Q38	Macroglossia	2.32%	1.45%	0.24	0.06

Patients matched by age, sex, and presence of diabetes mellitus.

*SD* standard difference.

To shed further light on the racial differences in AL amyloidosis, we examined the extent to which pre-existing diagnoses differ by race among patients with and without AL amyloidosis. Table 2 shows the results of this analysis applied to samples matched by age, sex, and presence of type 2 diabetes, which yielded 695 Black patients and 3333 White patients without AL amyloidosis. As expected, there was a significantly higher incidence of MGUS, as well as most other AL-associated organ-specific and other pre-existing diagnoses of interest preceding the diagnosis of AL amyloidosis within each racial group, the prevalence of which was considerably higher among Black patients with AL. This pattern confirms the notion that AL patients have a different clinical profile than those who do not develop the disease. These findings also suggest that it may be possible to develop an early warning system through advanced statistical models that would identify patients at risk for developing AL amyloidosis earlier in the disease course. Additionally, there was a higher prevalence of AL-associated pre-existing conditions among the subgroup of African American/Black individuals without AL when compared to their White counterparts, pointing to a higher burden of comorbidities or a higher proportion of undiagnosed AL amyloidosis in Black individuals compared to Whites. Staron, et al. recently reported that among patients seen at an Amyloid Center of Excellence, Black patients had a more aggressive phenotype^10^. Additionally, other systemic barriers and social determinants of health may further result in delays/exclusion of AL amyloidosis patients being referred to centers of excellence, thus resulting in additional race-based disparities in AL amyloidosis care, which needs to be further explored.

**Table 2:** Comparison between pre-existing diagnoses in individuals with or without AL amyloidosis stratified by race.

ICD10 code	Pre-existing diagnosis	Black, with AL *N* = 695	Black, no AL *N* = 695	*p*-value Black, AL vs not	SD	White, with AL *N* = 3333	White, no AL *N* = 3333	*p*-value White, AL vs not	SD
D47.2	MGUS	21.01%	1.44%	6.84E−31	0.65	16.41%	0.36%	0	0.61
C90	Multiple myeloma	32.23%	1.44%	0	0.90	32.67%	0.30%	0	0.97
***Cardiac diagnoses***									
I51.7	Cardiomegaly	28.92%	9.35%	1.81E−20	0.51	20.13%	4.08%	0	0.51
I42	Cardiomyopathy	37.41%	5.04%	0	0.86	23.19%	2.70%	0	0.64
I49	Other cardiac arrhythmia	30.50%	10.50%	2.59E−20	0.51	22.05%	8.16%	0	0.40
R06.0	Dyspnea	50.07%	21.73%	3.24E−28	0.62	38.52%	15.00%	0	0.55
R55	Syncope and collapse	13.53%	7.05%	7.1E−05	0.21	10.59%	3.93%	1.08E−25	0.26
R42	Dizziness and giddiness	14.53%	13.09%	0.43694	0.04	15.51%	9.57%	2.43E−13	0.18
R60	Edema	33.67%	12.95%	6.53E−20	0.51	27.69%	7.56%	0	0.55
I50	Heart failure	54.96%	11.94%	0	1.03	33.00%	5.88%	0	0.73
J90	Pleural Effusion	11.66%	2.16%	2.92E−12	0.38	10.08%	1.26%	0	0.39
I48	Atrial fibrillation and flutter	22.01%	6.04%	1.01E−17	0.47	21.84%	8.40%	0	0.38
I95	Hypotension	22.01%	6.33%	5.18E−17	0.46	17.91%	3.57%	0	0.48
***Renal diagnoses***									
N04	Nephrotic syndrome	8.35%	1.44%	2.39E−09	0.32	10.08%	0.30%	0	0.45
R80	Proteinuria	23.31%	4.17%	3.70E−25	0.58	19.92%	2.13%	0	0.59
N18	Chronic Kidney disease	43.89%	14.53%	2.38E−33	0.68	33.15%	6.48%	0	0.71
**Neurological diagnoses**									
N52.9	Male erectile dysfunction	7.63%	9.07%	0.332133	0.05	4.50%	4.71%	0.68	0.01
M79.2	Neuralgia and Neuritis	2.30%	3.31%	0.255555	0.06	3%	1.29%	1.45E−06	0.12
R20	Disturbances of skin sensations	13.96%	8.49%	0.001	0.17	14.13%	7.26%	1.13E−19	0.22
G60	Hereditary and idiopathic neuropathy	12.81%	3.02%	1.41E−11	0.37	10.77%	2.10%	0	0.36
G62	Other and unspecified polyneuropathies	11.66%	4.17%	2.38E−07	0.28	12.51%	3.78%	8.26E−39	0.32
G90	Disorders of autonomic nervous system	2.73%	1.44%	0.091225	0.09	3.24%	0.30%	8.82E−20	0.22
**GI/hepatic**									
R13.1	Dysphagia	12.81%	6.19%	2.57E−05	0.23	9.84%	5.19%	5.99E−13	0.18
R16	Hepatomegaly and splenomegaly	5.76%	1.73%	7.57E−05	0.21	4.35%	1.56%	1.74E−11	0.17
K76	Other diseases of liver	13.38%	6.91%	6.39E−05	0.22	9.15%	4.56%	1.21E−13	0.18
R11	Nausea and vomiting	20%	11.66%	2.02E−05	0.23	15.51%	7.29%	4.61E−26	0.26
K59.0	Constipation	22.16%	12.81%	4.43E−06	0.25	14.67%	6.45%	9.31E−28	0.27
R19.7	Diarrhea	15.54%	6.76%	2.01E−07	0.28	15.27%	6.90%	1.37E−27	0.27
R10	Abdominal pain	27.63%	24.60%	0.20	0.07	20.16%	17.01%	0.001	0.08
**Other**									
R53	Malaise and fatigue	30.79%	18.56%	1.24E−07	0.29	32.73%	15.12%	0	0.42
G56.0	Carpal Tunnel Syndrome	11.08%	4.17%	1.23E−06	0.26	8.34%	3.36%	4.60E−18	0.21
D69	Purpura	15.25%	3.45%	4.23E−14	0.41	12.90%	2.82%	0	0.38
K14.8	Macroglossia	2.30%	0%	5.74E−05	0.22	1.44%	0.30%	5.40E−07	0.12

Patients matched by age, sex, and presence of diabetes mellitus.

*SD* standard difference.

Our study is limited by the lack of granular data on AL amyloidosis, including the lack of information on stage and biomarkers. Nonetheless, these large, nationwide EHR data provide a valuable opportunity to understand the clinical burden of AL amyloidosis. These data also suggest that AL is not as rare even with our conservative definition using diagnostic codes. Our findings are consistent with the hypothesis that Black patients may experience under- or delayed diagnosis, as suggested by their higher burden of pre-existing, AL-associated diagnoses prior to the formal AL diagnosis. This is especially true for critical organ conditions associated with early mortality. Finally, there are significant differences, overall and across racial groups, in the prevalence of pre-existing diagnoses in individuals with and without AL, suggesting the feasibility and value of developing predictive algorithms aimed at identifying patterns of precursor conditions associated with the likelihood of an AL amyloidosis diagnosis. These results highlight significant racial disparities in AL amyloidosis diagnosis. Our next steps include studying differences in the length of time for pre-existing diagnoses prior to AL diagnosis as well outcomes after the diagnosis, including the burden of illness. Future work is also needed to study the causes and consequences of racial disparities on the disease course and mortality of AL amyloidosis patients.
